# Unbiased Estimates Using Temporally Aggregated Outcome Data in Time Series Analysis: Generalization to Different Outcomes, Exposures, and Types of Aggregation

**DOI:** 10.1097/EDE.0000000000001923

**Published:** 2025-10-02

**Authors:** Xavier Basagaña, Joan Ballester

**Affiliations:** From the a ISGlobal, Barcelona, Spain; b Universitat Pompeu Fabra (UPF), Barcelona, Spain; c CIBER Epidemiología y Salud Pública, Madrid, Spain

**Keywords:** Air pollution, Distributed lag nonlinear model, Temperature, Temporal aggregation, Time series, Monthly data, Mortality, Weekly data

## Abstract

**Background:**

A new method for time series analysis was recently formulated and implemented that uses temporally aggregated outcome data to generate unbiased estimates of the underlying association between temporally disaggregated outcome and covariate data. However, the performance of the method was only tested in the context of the delayed nonlinear relation between temperature and mortality, and only in the case of the aggregation of sets of consecutive days.

**Methods:**

We conduct a simulation analysis to test the performance of the method using (1) mortality and hospital admissions as health outcomes, (2) temperature and nitrogen dioxide as exposures, and (3) the three aggregation schemes most widely used in open-access health data, including aggregations of sets of nonconsecutive days.

**Results:**

With sufficient data for analysis, the method can recover the underlying association for all combinations of outcomes, exposures, and aggregation schemes. The bias and variability of the estimates increase with the degree of aggregation of the outcome data, and they decrease with increasing sample size (length of dataset, number of cases). Remarkably, estimates are also unbiased even in extreme cases with weekly outcome data in an association confounded by the day of the week, such as those of air pollution models.

**Conclusions:**

With sufficient data, the method is able to flexibly generate unbiased estimates, generalizing previous results to other outcomes, exposures, and types and degrees of aggregation. Such results can boost the use of available temporally aggregated health data for research, translation, and policymaking, especially in low-resource and rural areas.

Time series studies have extensively documented short-term associations between environmental exposures (e.g., temperature, air pollution) and health outcomes (e.g., mortality, morbidity).^1–3^ The requirement of having daily health data to conduct these studies has, however, restricted most of the empirical evidence to high-income urban settings. In a recent paper, Basagaña and Ballester^4^ developed a method with the capacity to circumvent this requirement, as it provides unbiased estimates of the association between temporally disaggregated exposure and temporally disaggregated response data, but only by requiring temporally disaggregated exposure and temporally aggregated (e.g., weekly, monthly) response data. The method opened a new avenue for research, translation, and policy making in low-income and rural areas, where temporally aggregated health data is often the only available option, especially in open-access format.

The original paper provided the theoretical basis for the method, and empirically tested its properties by using simulations and real multi-country data applied to the highly parametrized, delayed, nonlinear association between ambient temperature and all-cause mortality.^4^ Moreover, the article only tested the performance of two degrees of aggregation, with weekly and monthly outcome data, both representing aggregations of sets of consecutive days. In the present paper, we extend the simulation analysis by testing the performance of the method applied to the four combinations between two health outcomes (all-cause mortality and respiratory hospitalizations) and two environmental exposures (ambient temperature and nitrogen dioxide, or NO_2_, a marker of traffic-related air pollution in cities). These four cases represent (1) highly parametrized U-shaped associations with delayed effects up to 21 days (i.e., temperature with mortality or hospitalizations) and (2) much less parametrized linear associations with no or very short lagged effects (i.e., NO_2_ with mortality or hospitalizations). In addition, for each of these combinations, we test the performance of the method using the original daily health data and the three most common types of available aggregated health data, namely weekly,^5^ monthly,^6^ and groups of nonconsecutive days corresponding to the same day of the week, month and year (e.g., all Mondays of January 2025).^6^

## METHODS

### Data

We based simulations on data from the province of Barcelona (northeastern Spain) for the years 1995–2019. Daily counts of all-cause mortality and emergency hospital admissions from respiratory causes (International Classification of Diseases, 10th revision, ICD-10 codes: J00-J99) for all ages were obtained from the Spanish National Institute of Statistics. The daily average of all-cause mortality was 120 deaths, while the daily average of respiratory hospitalizations was 155 admissions. We obtained hourly gridded 2-m temperature data from the high-resolution European Centre for Medium-Range Weather Forecasts ReAnalysis 5 Land reanalysis,^7^ from which we calculated a single value of average temperature per day for the entire province. For NO_2_, we obtained hourly values from three official monitoring stations (Eixample, Poblenou, and Sants) of the Catalan Government, located in the city of Barcelona.^8^ We imputed missing values with the method of chained equations and with linear interpolation when the three stations were missing, and averaged the values of the three stations by day to obtain average daily concentrations. The health data used represent aggregated series, and no ethical review was required.

### Epidemiological Models

We separately fitted epidemiologic models for the associations between (1) temperature and mortality, (2) temperature and hospitalizations, (3) NO_2_ and mortality, and (4) NO_2_ and hospitalizations. We fitted quasi-Poisson regression models to these original datasets, using mortality or hospitalization counts as outcome variables. In addition to model-specific terms (see next paragraph), all models included an intercept and a natural cubic spline of time with 8 degrees of freedom per year to control for seasonal and long-term trends.

Both for mortality and hospitalizations, we obtained the results for temperature from a model that included a crossbasis function to estimate the exposure–lag–response association. On the one hand, we modeled the exposure–response association of the crossbasis with a natural cubic spline (ns), with three internal knots placed at the 10th, 75th, and 90th percentiles of daily temperatures. On the other hand, we modeled the lag–response association of the crossbasis with a natural cubic spline, with an intercept and three internal knots placed at equally spaced intervals in the log scale, with lags ranging between 0 and 21 days. The equation of the temperature models for both mortality and hospitalizations (outcome) was


$$\begin{array}{l} \begin{array}{lll} log\left(E\left(outcome\right)\right) & =intercept+ns\left(time,8dfperyear\right) & +crossbasis\left(temperature,\;0\text{–}21days\right). \end{array} \end{array}$$

We obtained the results for NO_2_ from a model that included a linear term averaging NO_2_ for the current and previous day (lag 0–1), divided by 20 so that the regression models estimate the effect for a 20 μg/m^3^ increase in NO_2_ concentration (value similar to the interquartile range). Importantly, the NO_2_ models included dummy variables for day of the week (dow) and holidays, which are well-known confounders of the relation between air pollution and mortality or hospitalizations. Models also included separate additional terms to control for high and low temperatures, according to previous approaches used in air pollution studies.^9^ For high temperatures, we used the timeseries defined by the average temperature of the current and previous day (lag 0–1) for days warmer than the median temperature and the median temperature for days colder than the median temperature. For low temperatures, we used instead the timeseries defined by the average temperature of the previous 6 days (lag 1–6) for days colder than the median temperature, and the median temperature for days warmer than the median temperature. We here refer to these variables as *temp_lag01_heat* and *temp_lag16_cold*, respectively. Then, *temp_lag01_heat* was included in the model with a natural cubic spline with knots at the 75th and 90th percentiles, and *temp_lag06_cold* with a natural cubic spline with a knot at the 10th percentile. The equation of the NO_2_ models for both mortality and hospitalizations was


$$\begin{array}{l} \begin{array}{llll} log\left(E\left(outcome\right)\right) & =intercept+ns\left(time,8dfperyear\right)+NO_{2}\left(0-1days\right) & +ns\left(temp\_lag01\_heat\right)+ns\left(temp\_lag16\_cold\right) & +dow_{}+holidays. \end{array} \end{array}$$

### Simulation Analyses

For each exposure–outcome pair and aggregation scheme, we conducted simulations using 2, 3, …, 9, 10, 15, 20, and 25 years of data. The methodology used in the simulations is described in detail in the eMethods; https://links.lww.com/EDE/C280. In a nutshell, we used a negative binomial distribution to simulate the mortality or hospitalization counts, whose mean was the prediction of the model regression equations described in the previous subsection, but with specific values of the parameters. The values of the parameters were taken from the regression model using the original daily exposure and daily outcome data, as described in the eMethods; https://links.lww.com/EDE/C280 to use realistic values. eFigures 1–2; https://links.lww.com/EDE/C280 display the exposure-response and lag-response functions used to simulate data for the temperature models. The linear term of NO_2_ took the value 0.005 in the mortality-NO_2_ models, while it was set to 0.010 in the hospitalizations-NO_2_ models. The former value was obtained from the regression model using the original daily exposure and daily outcome data, while the latter was instead chosen within the range of associations for NO_2_ reported in previous studies^10^ to test a scenario with higher statistical power (the value obtained from the original daily exposure and daily outcome data was equal to 0.0025, see eMethods; https://links.lww.com/EDE/C280).

We simulated 500 outcome time series for each pair of exposure–outcome variables and length of the dataset. Then, each simulated dataset was analyzed with the usual quasi-Poisson regression model (see equations in the previous subsection) to obtain the estimates corresponding to daily exposure and simulated daily health data (Daily|Daily, or D|D). In addition, we applied the method developed by Basagaña and Ballester^4^ (see eMethods; https://links.lww.com/EDE/C280) to daily exposure and the three types of aggregated simulated health data: (1) weekly (weekly|daily, W|D), (2) monthly (monthly|daily, M|D), and (3) groups of nonconsecutive days corresponding to the same day of the week, month and year (Dow|D). For some models, for example, W|D models for NO_2_, where weekly health data is adjusted for day of the week, estimation of the models resulted in rank-deficient Hessian matrices. In those cases, to estimate standard errors of the parameters, we computed a generalized covariance matrix as the generalized Moore–Penrose inverse of the negative of the Hessian matrix.^11^

For the temperature models, we calculated the bias, root mean squared error (RMSE), and coverage of 95% confidence intervals (CIs) for the cumulative exposure–response association (in the log scale). For these calculations, we centered the association at the minimum mortality temperature obtained from the analysis of the original (i.e., nonsimulated) data, calculated the bias, RMSE, and coverage at each temperature percentile, and then averaged each of the indicators for the whole range of percentiles, following similar simulation studies.^4,12^ In addition, we calculated the statistical power at 5% significance level at the 1st and 99th temperature percentiles. For the NO_2_ models, which involved the analysis of the performance of a single coefficient, we calculated the bias, RMSE, coverage of 95% CIs, and statistical power at 5% significance level as customarily done in simulation studies.^13^

## RESULTS

Figure panels A–D and E–H show the performance of the four aggregation models for temperature with mortality and hospitalizations. Results show unbiased estimates for any length of the dataset in D|D and W|D, absence of bias with 5 or more years of data in Dow|D, and rapidly decreasing bias magnitude with increasing dataset length in M|D (Figure A,E). The RMSE, which in all cases monotonically decreases with the length of the dataset, is only minimally higher in W|D than D|D, somewhat larger in Dow|D, and substantially higher in M|D (Figure B,F). The coverage in D|D, W|D, and Dow|D was at the nominal value, while M|D suffered from an under-coverage that rapidly decreased in magnitude with the number of years of the dataset (Figure C,G). Results for statistical power were aligned with those of RMSE, in the sense that W|D had only slightly lower power than D|D, whereas Dow|D, and especially M|D, exhibited substantially lower power (Figure D,H). eFigures 3–9; https://links.lww.com/EDE/C280 display the same results for each temperature percentile. As expected, the largest biases and RMSE occurred at extreme temperatures, although they also rapidly decreased with the number of years of the dataset (eFigures 3–5; https://links.lww.com/EDE/C280). Instead, coverage was generally well-behaved at all temperature percentiles (eFigures 6–9; https://links.lww.com/EDE/C280).

**FIGURE:**
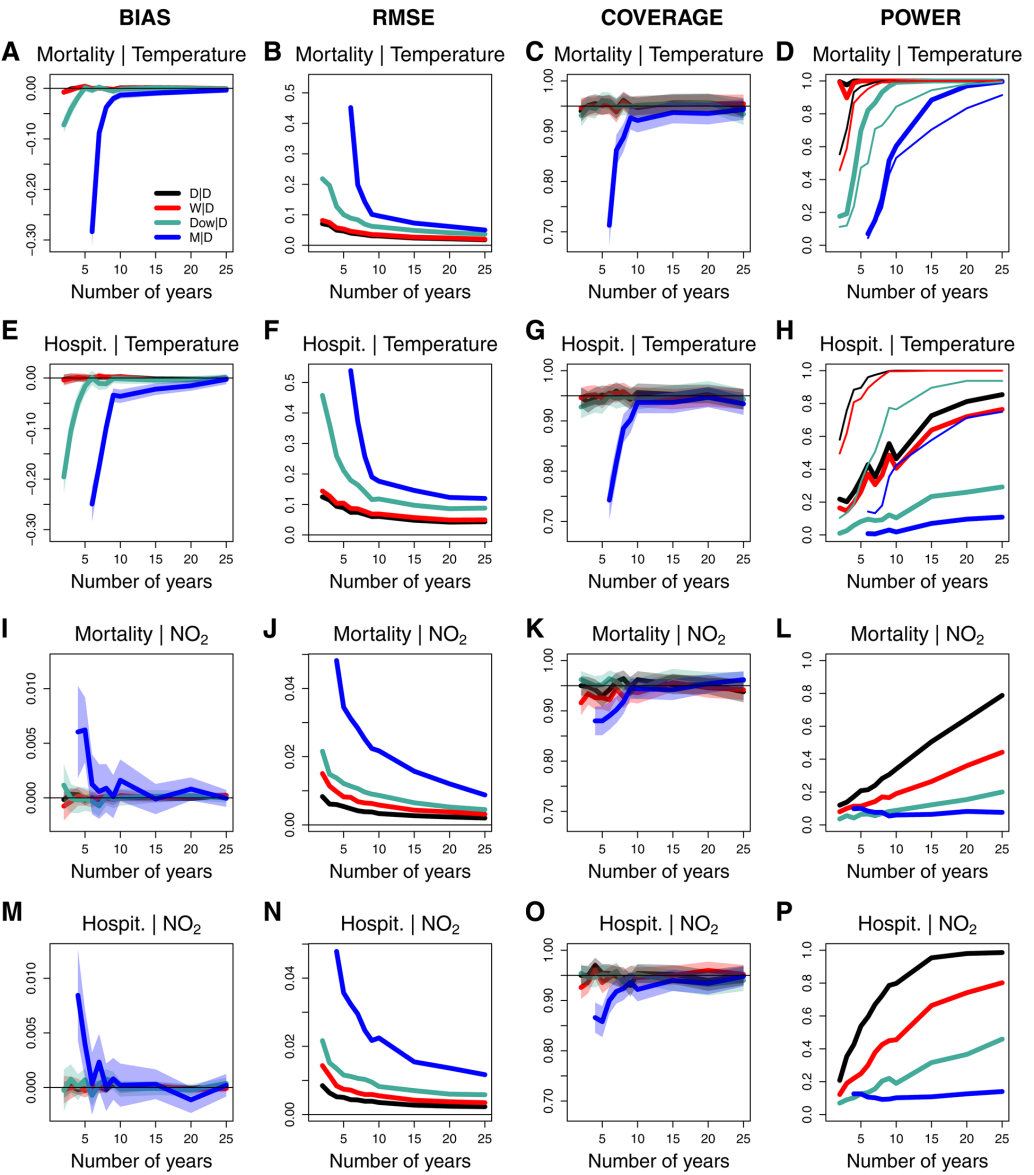
Bias, root mean squared error, coverage, and statistical power of the cumulative exposure–response association. Average bias (A,E,I,M), RMSE (B,F,J,N), coverage of 95% confidence intervals (C,G,K,O), and statistical power at 5% significance level (D,H,L,P) of the exposure–response association as a function of the length of the dataset. For temperature (A–H), we calculated these metrics from the cumulative exposure–response function, computed from the logarithm of the relative risks and centered at the minimum mortality temperature of the true association. We calculated the statistical power for detecting the association at the 1st (thin lines) and 99th (thick lines) percentiles of the daily temperature distribution (D,H), or for the single regression coefficient of NO_2_ (L,P). Lines represent performance values in the Daily|Daily (D|D, black), Weekly|Daily (W|D, red) and Monthly|Daily (M|D, blue) models, as well as for groups of days corresponding to the same day of the week, month, and year (e.g. all Mondays of January 2025; Dow|D, green). Shaded areas in the bias and coverage correspond to 95% confidence intervals.

Figure I–P show the performance for NO_2_. In all cases, estimates were unbiased, except for M|D with less than 5 years of data (Figure I,M). We found the lowest RMSE for D|D models, followed by W|D, Dow|D, and M|D, the latter with much higher values (Figure J,N). The coverage of all models was at the nominal level, except for some under-coverage of M|D when using less than 10 years of data (Figure K,O). The statistical power confirms the results observed for RMSE, with decreases in precision when using aggregated data, causing important reductions in statistical power (Figure L,P).

As expected, worse performance results are observed when the number of deaths or hospitalizations is divided by 10 (eFigure 10; https://links.lww.com/EDE/C280).

## DISCUSSION

We here showed that, regardless of the outcome variable considered, the estimation method is able to provide unbiased estimates of the parameters of the temporally disaggregated model when enough data is included in the analyses. As expected, temporal aggregation comes with reductions in precision and, consequently, in statistical power.

The results of this analysis extend the conclusions of the original paper to a wider range of cases, including (1) different outcomes with different exposure–lag–response associations; (2) exploring the case of air pollution, with an association defined by a single parameter; and (3) testing the aggregation method that combines year, month and day of the week (Dow|D models), which was not tested before and uses sets of nonconsecutive days. Despite consisting of temporal aggregations of sets of only 4 or 5 days, Dow|D models performed worse than W|D (with aggregations of 7 days), showing that nonconsecutive aggregation schemes generally perform worse, although they were still able to substantially improve the performance of M|D. This result proves the capacity of the method to flexibly recover underlying delayed nonlinear associations if enough data is used, even when only temporally aggregated outcome data for sets of nonconsecutive days is available.

Of note, we showed that the estimation method is able to correct for confounding by day of the week and holidays even when the outcome data is temporally aggregated in the W|D, Dow|D, and M|D models. Remarkably, the W|D model generates unbiased estimates of the relation between air pollution and outcome, which is strongly confounded by the weekly cycle, thanks to the fact that the day of the week pattern is available in the exposure. Model fitting in such extreme cases results in rank-deficient Hessian matrices, but the simulations show that computing standard errors via a generalized covariance matrix produced CIs with nominal coverage.

Temporal aggregation of health data comes with a reduction of statistical power that implies that, to achieve the same power as in a disaggregated analysis, the length of the dataset should be extended. Although this is context-dependent, we observed that for the case of temperature, W|D models required almost no length extension in comparison with D|D models. For example, to detect the effects of cold on mortality with 80% power, D|D and W|D models required less than 4 years of data, while Dow|D and M|D required 10 and 20 years, respectively. In the models for hospitalizations and NO_2_, models achieved 80% power with 10 and 25 years for the D|D and W|D models, respectively, while power was still low with 25 years for Dow|D and especially M|D.

Overall, these new results generalize those of the original article and exemplify how the method can be applied to other outcomes, exposures, and types of aggregation. This method can boost the use of available temporally aggregated data for research purposes, not only in the field of public health but also in other fields using time series data.^14–16^

## ACKNOWLEDGMENTS

J.B. gratefully acknowledge funding from the European Union’s Horizon 2020 and Horizon Europe research and innovation programmes under grant agreement No 865564 (European Research Council Consolidator Grant EARLY-ADAPT, https://www.early-adapt.eu/), 101069213 (European Research Council Proof-of-Concept HHS-EWS, https://forecaster.health/), and 101123382 (European Research Council Proof-of-Concept FORECAST-AIR), and from the Spanish Ministry of Science and Innovation under grant agreement No RYC2018-025446-I (programme Ramón y Cajal). Both authors acknowledge funding from the Ministry of Research and Universities of the Government of Catalonia (2021-SGR-01563), support from the grant CEX2023-0001290-S funded by MCIN/AEI/10.13039/501100011033, and support from the Generalitat de Catalunya through the CERCA Program.

## Supplementary Material


