# Supplementary material for: Unbiased Estimates Using Temporally Aggregated Outcome Data in Time Series Analysis: Generalization to Different Outcomes, Exposures, and Types of Aggregation
Source: Epidemiology. 2025 Oct 2;37(1):16–20. doi: 10.1097/EDE.0000000000001923 (PMC12643558; doi:10.1097/EDE.0000000000001923)
Supplement: Supplementary file 1 [file ede-37-016-s001.pdf]

Unbiased estimates using temporally aggregated outcome data in timeseries analysis: generalization to different outcomes, exposures and types of aggregation – eAppendix

eMethods

Brief explanation of the estimation method used

Basagaña and Ballester (2024) originally derived the method to estimate the parameters of the underlying temporally disaggregated model when only temporally aggregated outcome data and temporally disaggregated exposure data are provided. We assume the following Poisson regression model for the temporally disaggregated data,

$$Y_t | X_t \sim Poisson\left(\exp\left(\sum_{k=1}^{K} \beta_k X_{t,k}\right)\right),$$

where Y_t is the outcome variable at time *t*, X_t is a matrix of covariates (including those related with the exposure of interest), β_k is the coefficient associated with the k-th covariate $X_{t,k}$, and $t = 1, \dots, T$. Then, suppose the study period is partitioned into non-overlapping temporal aggregations $\alpha_1, \dots, \alpha_A$ of sizes $n_1, \dots, n_A$, with $T = \sum_{a=1}^{A} n_a$. Then, Basagaña and Ballester (2024) showed that the temporal aggregation of Y_t for $\alpha_1, \dots, \alpha_A$, $S_a = \sum_{t\in\alpha_a} Y_t$, follows a Poisson distribution,

$$S_a | \{X_t, \text{for all } t \in \alpha_a\} \sim Poisson\left(\sum_{t\in\alpha_a} \exp\left(\sum_{k=1}^{K} \beta_k X_{t,k}\right)\right).$$

Thus, one can build the likelihood function using only the values of S_a (temporally aggregated outcome), X_t (temporally disaggregated covariates, including the exposure of interest), and the parameters of the original temporally disaggregated model, β_k. Maximization of this likelihood function provides estimates of the original model parameters, β_k.

Description of the simulation study

We used temperature, nitrogen dioxide (NO_2), all-cause mortality and respiratory hospitalization data from the city of Barcelona to conduct separate simulation studies using 2, 3, 4, 5, 6, 7, 8, 9, 10, 15, 20 and 25 years of data. We always considered the period ending in December 31st 2019, e.g. the period 2018-2019 for the 2-year analysis, or 1995-2019 for the 25-year analysis.

Simulations for the different health outcomes and exposures were based on the following models.

For mortality and temperature, we used

log(E(mort)) = intercept + ns(time, 8 df per year) + crossbasis(temperature, 0-21 days), [eq. 1]

where *ns* is a natural cubic spline of time with 8 degrees of freedom per year to control for the seasonal and long-term trends, and the crossbasis function is used to estimate the exposure-lag-response association between daily temperatures (*temp*) and daily mortality counts (*mort*).

For respiratory hospitalizations and temperature, we similarly used

log(E(hosp)) = intercept + ns(time, 8 df per year) + crossbasis(temperature, 0-21 days), [eq. 2]

where *hosp* indicates the daily respiratory hospitalization counts.

For mortality and NO_2, we used

log(E(mort)) = intercept + ns(time, 8 df per year) + NO_2*(0-1 days) + ns(temp_lag01_heat) + ns(temp_lag16_cold) + dow + holidays,* [eq. 3]

where *dow* are six indicator variables for day of the week and *holi* is an indicator variable for holidays.

For respiratory hospitalizations and NO_2, we similarly used

log(E(hosp)) = intercept + ns(time, 8 df per year) + NO_2*(0-1 days) + ns(temp_lag01_heat) + ns(temp_lag16_cold) + dow + holi.* [eq. 4]

The true values of the parameters used to simulate new data were determined in the following way.

For the **temperature models**, [eq.1-2], for each health outcome, denoted as [*outcome*]:

1) To set the values of the parameters of the temperature crossbasis:

a) we calculated the overall mean (*avg_temp*$_{25}$) and standard deviation (*sd_temp*$_{25}$) of temperature using the data from the whole 25-year data period;

b) we took the dataset with 25 years of data and

i) fitted the corresponding model ([eq.1] when *outcome = mort*, or [eq.2] when *outcome = hosp*)

ii) stored the estimated parameters for the temperature crossbasis as *coef_true*[*outcome, temperature*], and the estimated dispersion parameters as *disp_par*[*outcome*, *temperature*].

2) To set the values of the parameters of the intercept and the spline of time, the following process was repeated for each length of the dataset, *i = 2, 3, ..., 9, 10, 15, 20, 25*:

a) we took the *i*-th dataset (corresponding to *i* years of data),

i) in order to remove any eventual effect of climate change, we standardized the *i*-th time series of temperature by defining

$$temp2_i(t) = (temp(t) - avg\_temp_i) * sd\_temp_{25} / sd\_temp_i + avg\_temp_{25};$$

ii) we fitted the model between $temp2_i$ and *outcome*, and stored the estimated parameters of the intercept and the spline of time as *coef_true_intercept*[*outcome*, *temperature*, *i*] and *coef_true_time*[*outcome*, *temperature*, *i*], respectively.

Then, in order to simulate new *outcome* data for the simulation study with *i* years of data:

1) we standardized the time series of temperature by defining

$$temp2_i(t) = (temp(t) - avg\_temp_i) * sd\_temp_{25} / sd\_temp_i + avg\_temp_{25};$$

2) we created a linear predictor, *lin_pred*[*outcome*, *temperature*, *i*], by combining the observed temperature and covariate data with the true coefficients *coef_true_intercept*[*outcome*, *temperature*, *i*], *coef_true_time*[*outcome*, *temperature*, *i*] and *coef_true*[*outcome*, *temperature*] (note that the latter is the same for all *i*); and

3) we simulated the outcome data from a negative binomial distribution with mean equal to λ = exp(*lin_pred*[*outcome, temperature*, *i*]) and variance equal to λ* *disp_par*[*outcome*, *temperature*].

The resulting exposure-lag-response functions that were used to simulate the data are shown in eFigure 1 for mortality and in eFigure 2 for respiratory hospitalizations.

Similarly, for the **NO_2 models**, [eq.3-4], for each health outcome, denoted as [*outcome*]:

1) To set the values of the parameters associated with NO_2:

a) we took the dataset with 25 years of data and

i) fitted the corresponding model ([eq.3] when *outcome = mort*, or [eq.4] when *outcome = hosp*)

ii) stored the estimated parameters for NO_2 as *coef_true*[*outcome,* NO_2], and the estimated dispersion parameters as *disp_par*[*outcome*, NO_2]. In addition, the estimated parameters for *ns*(*temp_lag01_heat*), *ns*(*temp_lag16_cold*), *dow* and *holi* were stored as *coef_true_temp_lag01*[*outcome*], *coef_true_temp_lag16*[*outcome*], *coef_true_dow*[*outcome*], and *coef_true_holi*[*outcome*].

2) To set the values of the parameters of the intercept and the spline of time, the following process was repeated for each length of the dataset, *i = 2, 3, ..., 9, 10, 15, 20, 25*:

a) we took the *i*-th dataset (corresponding to *i* years of data),

i) we standardized the time series of temperature by defining

$$temp2_i(t) = (temp(t) - avg\_temp_i) * sd\_temp_{25} / sd\_temp_i + avg\_temp_{25};$$

ii) we fitted the model, and stored the estimated parameters of the intercept and the spline of time as *coef_true_intercept*[*outcome*, NO_2, *i*] and *coef_true_time*[*outcome*, NO_2, *i*], respectively.

Then, in order to simulate new *outcome* data for the simulation study with *i* years of data:

1) we standardized the time series of temperature by defining

$$temp2_i(t) = (temp(t) - avg\_temp_i) * sd\_temp_{25} / sd\_temp_i + avg\_temp_{25};$$

2) we created a linear predictor, *lin_pred*[*outcome, NO_2, i*], by combining the observed temperature and covariate data with the true coefficients *coef_true_intercept*[*outcome, NO_2, i*], *coef_true_time*[*outcome, NO_2, i*], *coef_true*[*outcome, NO_2*], *coef_true_temp_lag01*[*outcome*], *coef_true_temp_lag16*[*outcome*], *coef_true_dow*[*outcome*], and *coef_true_holi*[*outcome*] (note that the last five terms are the same for all *i*); and

3) we simulated the outcome data from a negative binomial distribution with mean equal to λ = exp(*lin_pred*[*outcome, NO_2, i*]) and variance equal to λ* *disp_par*[*outcome, NO_2*].

Thus, the true values of all parameters were based on results obtained when analyzing the original data. We did an exception for *coef_true*[*outcome=hosp, NO_2*], given that the process described above produced a value of 0.0025. Since this value was too small to be detected with the sample size of the study, we replaced it by 0.010 to illustrate a situation with more statistical power. An effect size of 0.010 (i.e. a 1% increase per interquartile range increase) is plausible as it has been reported in the literature of short-term effects of air pollution.[1] For mortality, the value given to *coef_true*[*outcome=mort, NO_2*] was based on real data, i.e. 0.005.

Next, for each outcome and length of the dataset, we generated 500 simulated outcome vectors and fitted the D|D, W|D, Dow|D and M|D models.

Then, for the temperature effects, we calculated the bias, root mean squared error (RMSE) and coverage of the 95% confidence intervals at each temperature percentile of the cumulative exposure-response function, and then averaged these three indicators for the whole range of percentiles, following similar simulation studies.[2,3] Calculations involved the comparison of the estimated and true values of the logarithm of the relative risk (RR) at each temperature percentile, the latter calculated via *coef_true*[*outcome, temperature*]. In addition, we calculated the statistical power at 5% significance level at the 1st and 99th temperature percentiles.

For the NO_2 models, which have a single coefficient associated with the outcome variable, we calculated bias, root mean squared error (RMSE), coverage of the 95% confidence intervals and statistical power at 5% significance level as customarily done in simulation studies.[4]

The whole process was repeated by dividing the mean of the negative binomial distribution by 10, effectively reducing the daily number of cases by a factor of 10, to illustrate scenarios with smaller populations, namely cases with a daily average of 12 deaths and 15.5 hospitalizations.

The R codes to reproduce the simulations can be found at https://github.com/xbasagana/aggregated.

References

1 WHO. Review of evidence on health aspects of air pollution: REVIHAAP project: technical report. World Health Organization. Regional Office for Europe, 2013 https://iris.who.int/handle/10665/341712 (accessed Sept 27, 2024).

2 Gasparrini A. Modelling Lagged Associations in Environmental Time Series Data: A Simulation Study. *Epidemiology* 2016; **27**: 835–42.

3 Basagaña X, Ballester J. Unbiased temperature-related mortality estimates using weekly and monthly health data: a new method for environmental epidemiology and climate impact studies. *Lancet Planet Health* 2024; **8**: e766–77.

4 Morris TP, White IR, Crowther MJ. Using simulation studies to evaluate statistical methods. *Stat Med* 2019; **38**: 2074–102.

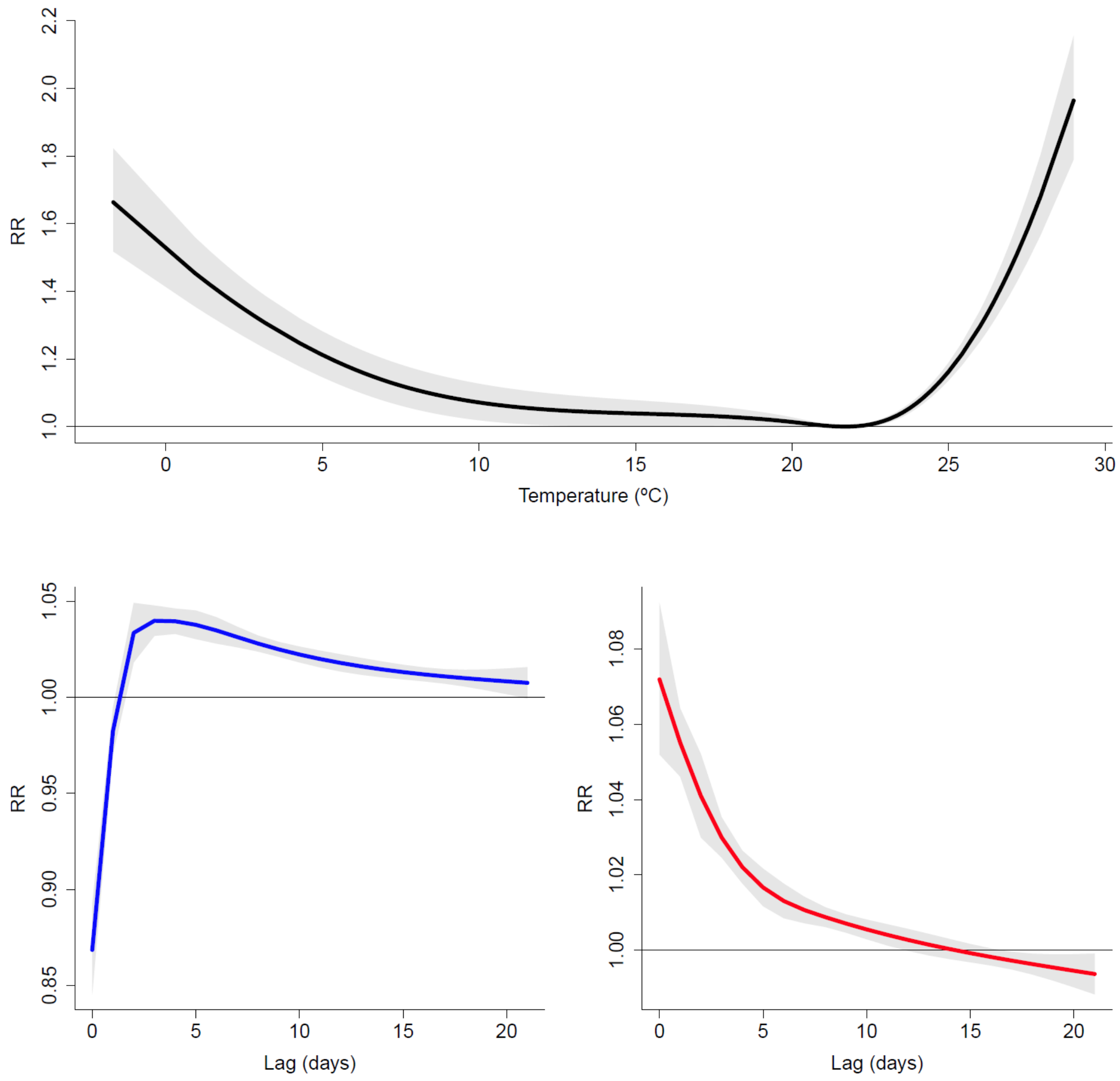

eFigure 1. Exposure-lag-response function for all-cause mortality and temperature in Barcelona (1995-2019).

Cumulative exposure-response (top) and lag-response functions at the 1st (bottom left) and 99th (bottom right) temperature percentiles. Shaded areas correspond to the 95% confidence intervals. RR = relative risk.

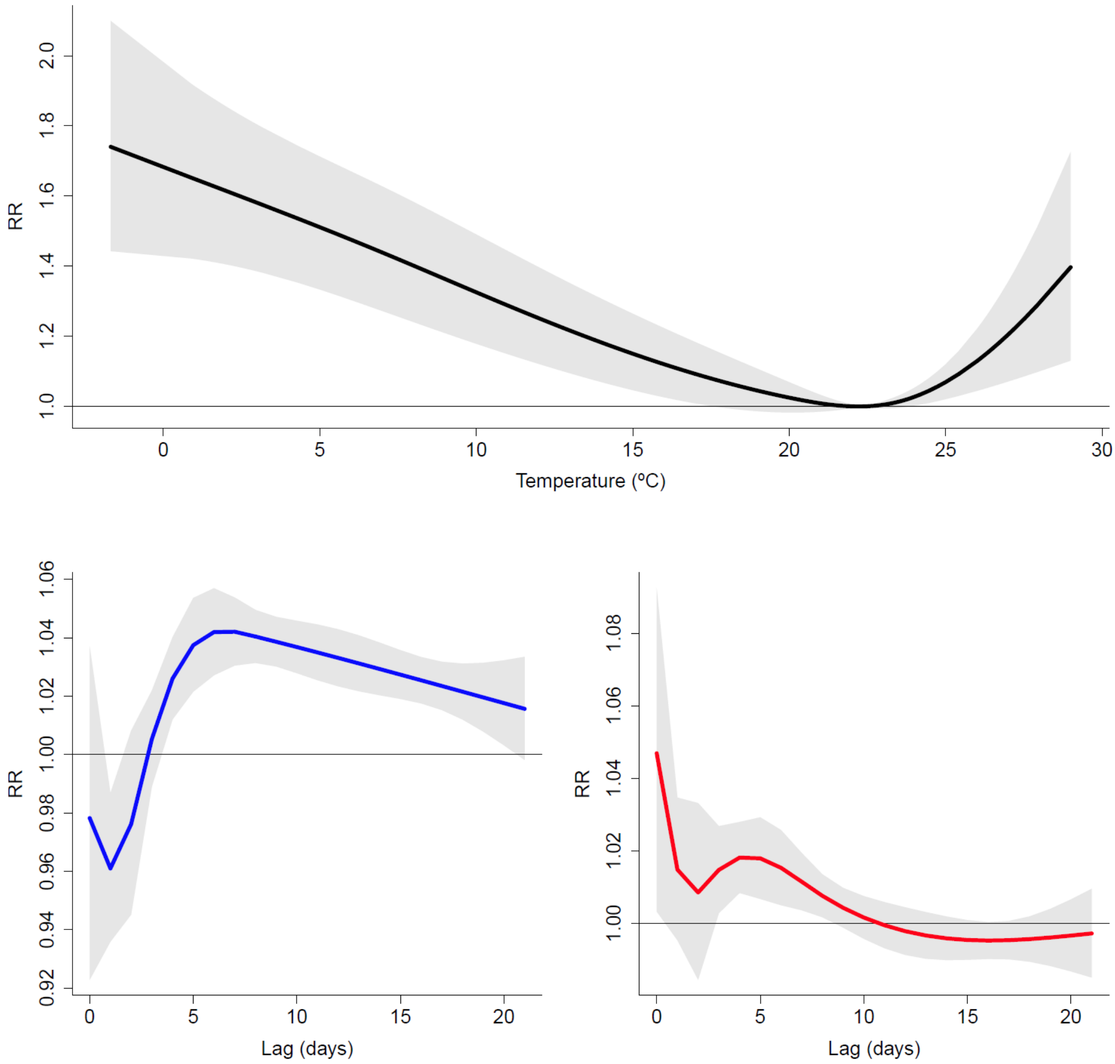

eFigure 2. Exposure-lag-response function for respiratory hospitalizations and temperature in Barcelona (1995-2019).

Cumulative exposure-response (top) and lag-response functions at the 1st (bottom left) and 99th (bottom right) temperature percentiles. Shaded areas correspond to the 95% confidence intervals. RR = relative risk.

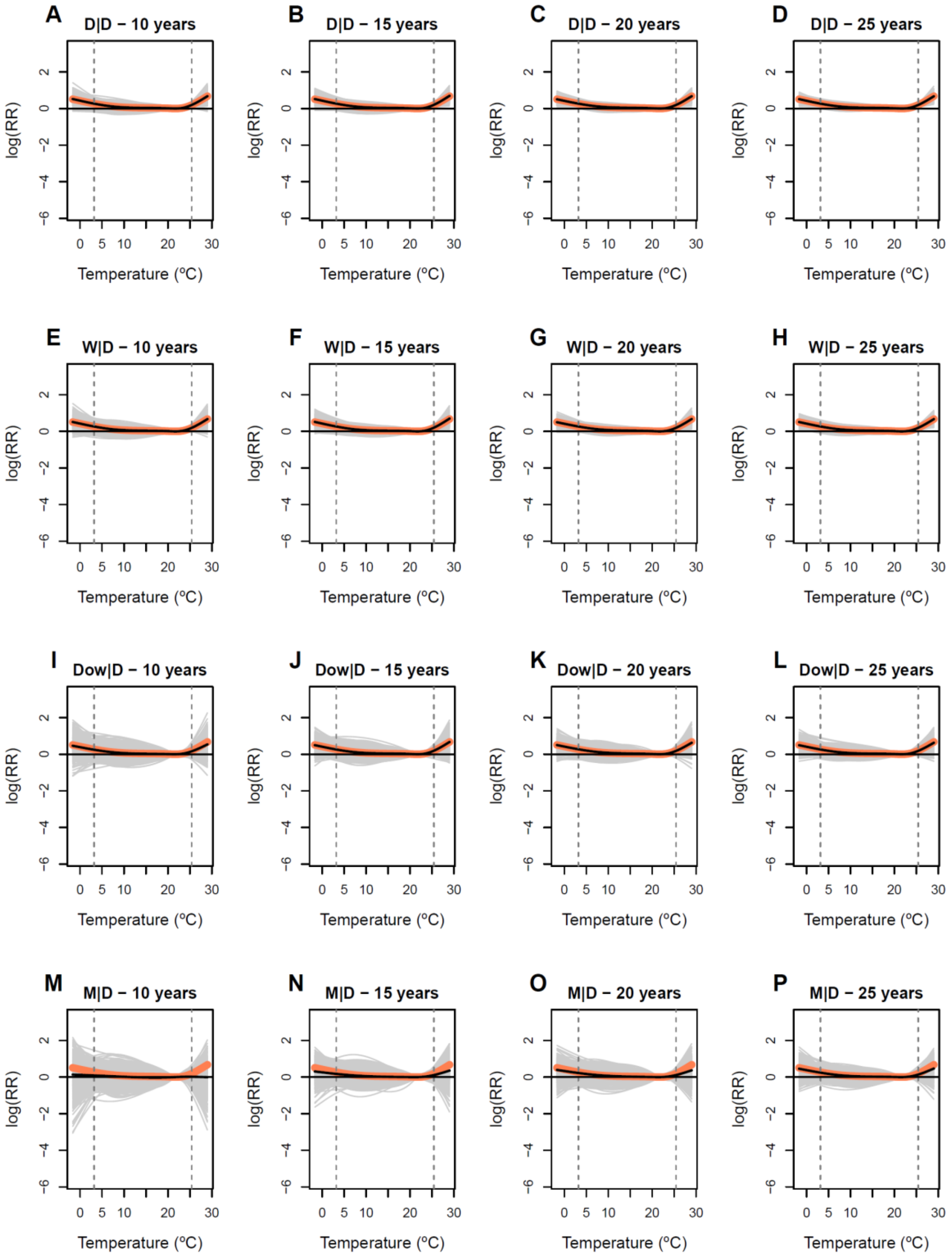

eFigure 3. Cumulative exposure-response association in the simulations for mortality and temperature.

Cumulative exposure-response associations, depicted for the true association (orange), each individual simulation (grey) and their average (black) in the D|D (A-D), W|D (E-H), Dow|D (I-L) and M|D (M-P) models. All the associations were centred at the minimum mortality temperature of the true association. Vertical dashed lines indicate the 1st and 99th temperature percentiles. Results for 10 years (A, E, I, M), 15 years (B, F, J, N), 20 years (C, G, K, O) and 25 years (D, H, L, P) are presented.

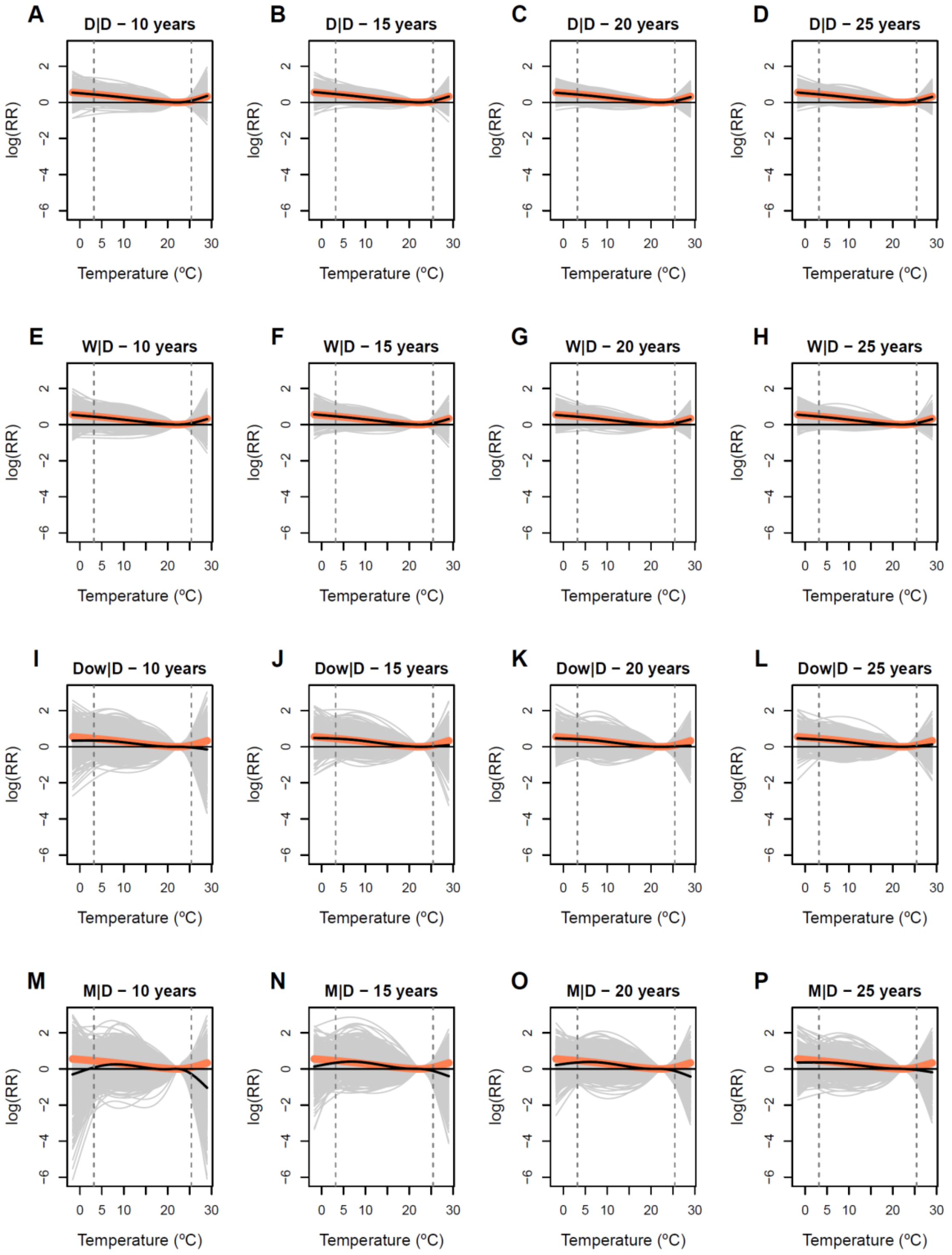

eFigure 4. Cumulative exposure-response association in the simulations for hospitalizations and temperature.

Cumulative exposure-response associations, depicted for the true association (orange), each individual simulation (grey) and their average (black) in the D|D (A-D), W|D (E-H), Dow|D (I-L) and M|D (M-P) models. All the associations were centred at the minimum hospitalization temperature of the true association. Vertical dashed lines indicate the 1st and 99th temperature percentiles. Results for 10 years (A, E, I, M), 15 years (B, F, J, N), 20 years (C, G, K, O) and 25 years (D, H, L, P) are presented.

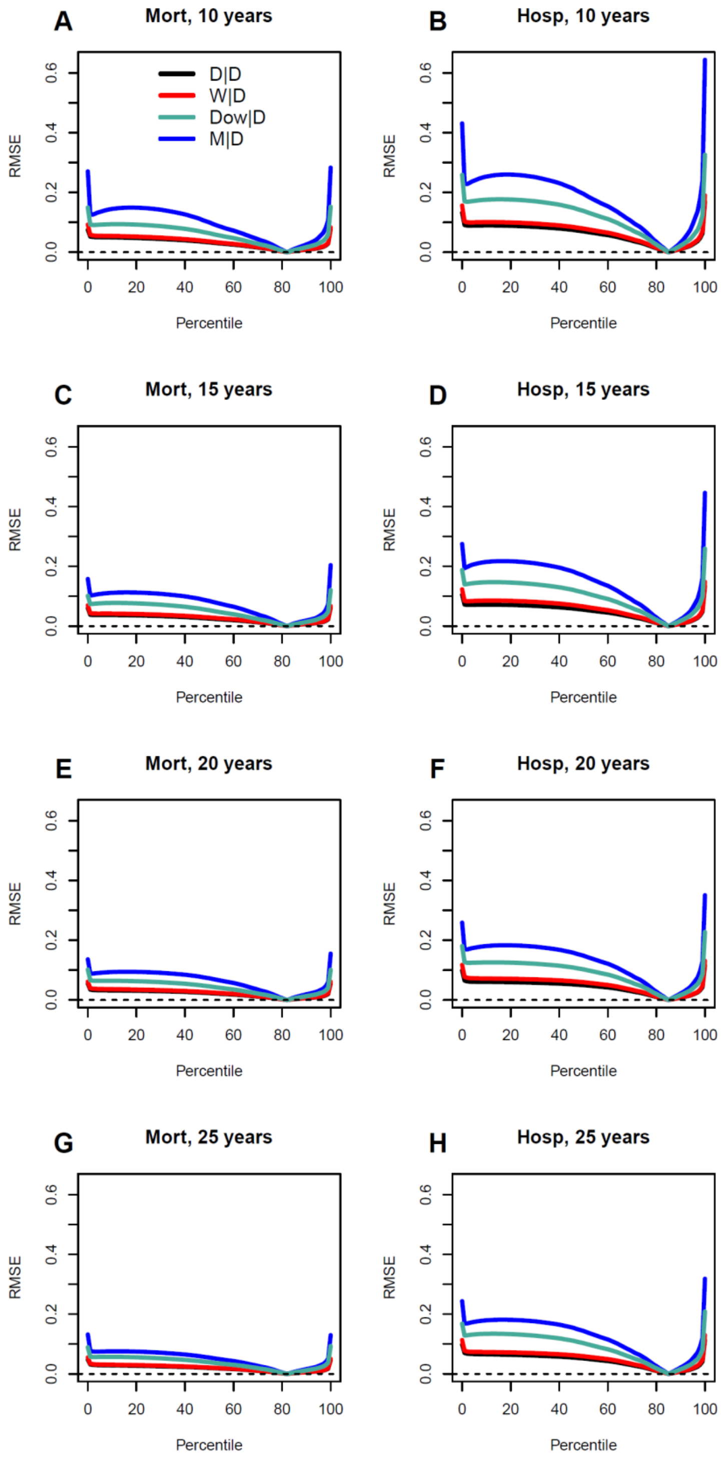

eFigure 5. Root mean square error (RMSE) of the cumulative exposure-response association at each temperature percentile.

Panels in the left column (A, C, E, G) show results obtained for mortality models, while panels in the right column (B, D, F, H) show results obtained for respiratory hospitalization models. Results for 10 years (A, B), 15 years (C, D), 20 years (E, F) and 25 years (G, H) are presented. Lines represent performance values in the Daily|Daily (D|D, black), Weekly|Daily (W|D, red) and Monthly|Daily (M|D, blue) models, as well as for groups of days corresponding to the same day of the week, month and year (e.g. all Mondays of January 2025, Dow|D, green).

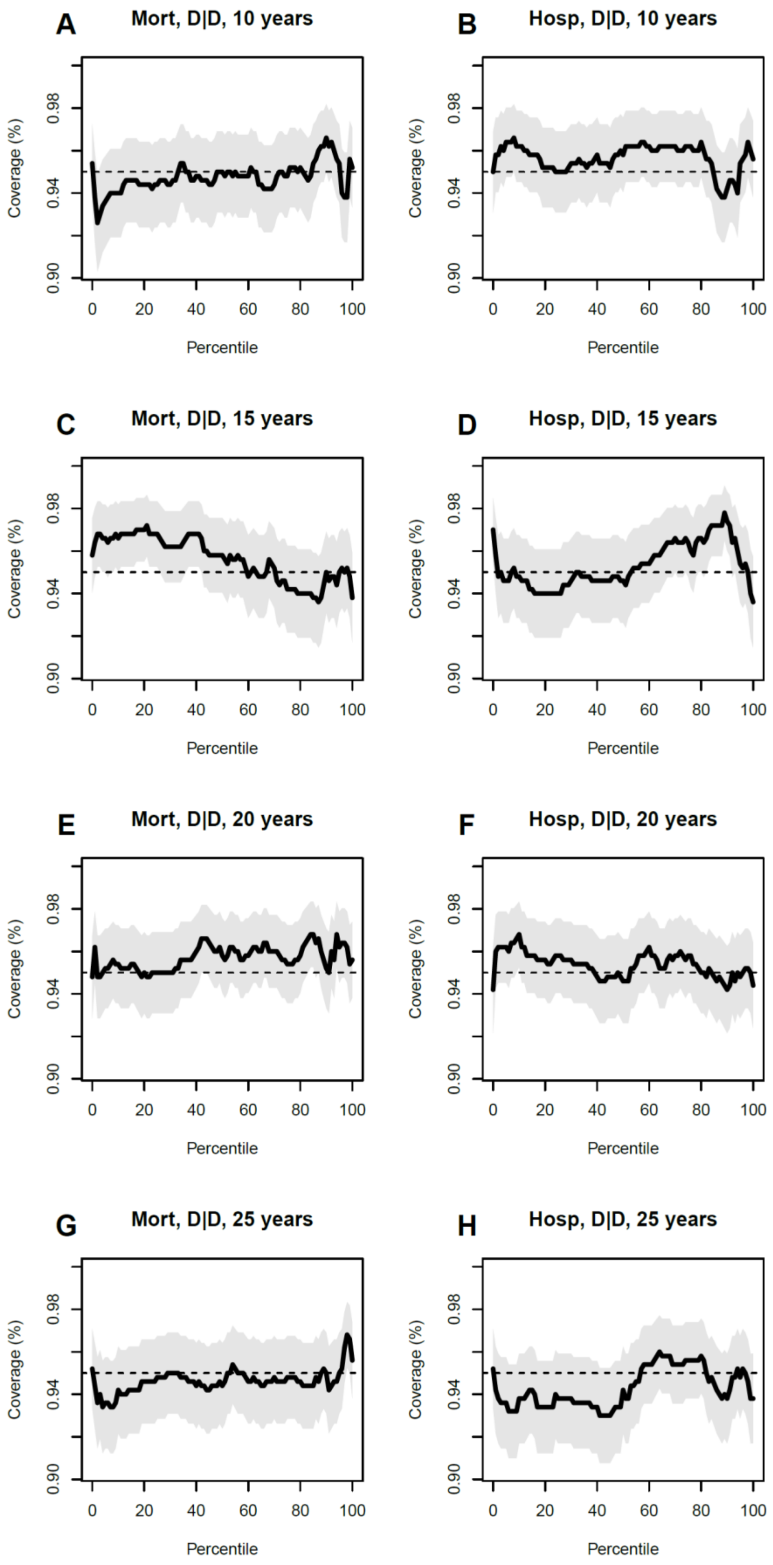

eFigure 6. Coverage of 95% confidence intervals at each temperature percentile (D|D model). Panels in the left column (A, C, E, G) show results obtained for mortality models, while panels in the right column (B, D, F, H) show results obtained for respiratory hospitalization models. Results for 10 years (A, B), 15 years (C, D), 20 years (E, F) and 25 years (G, H) are presented. Dashed lines correspond to the nominal 95% level. Shaded areas correspond to the 95% confidence intervals.

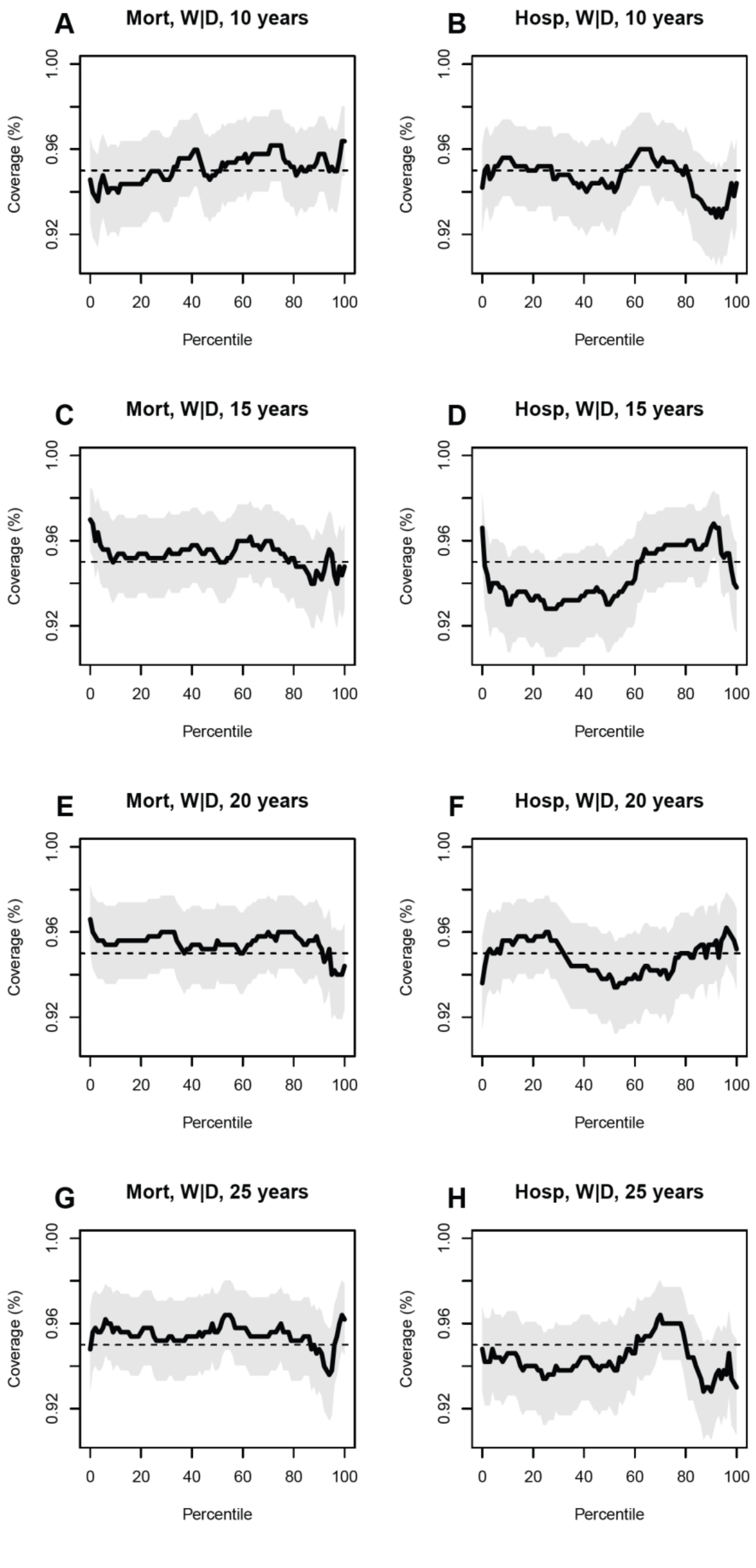

eFigure 7. Coverage of 95% confidence intervals at each temperature percentile (W|D model). Panels in the left column (A, C, E, G) show results obtained for mortality models, while panels in the right column (B, D, F, H) show results obtained for respiratory hospitalization models. Results for 10 years (A, B), 15 years (C, D), 20 years (E, F) and 25 years (G, H) are presented. Dashed lines correspond to the nominal 95% level. Shaded areas correspond to the 95% confidence intervals.

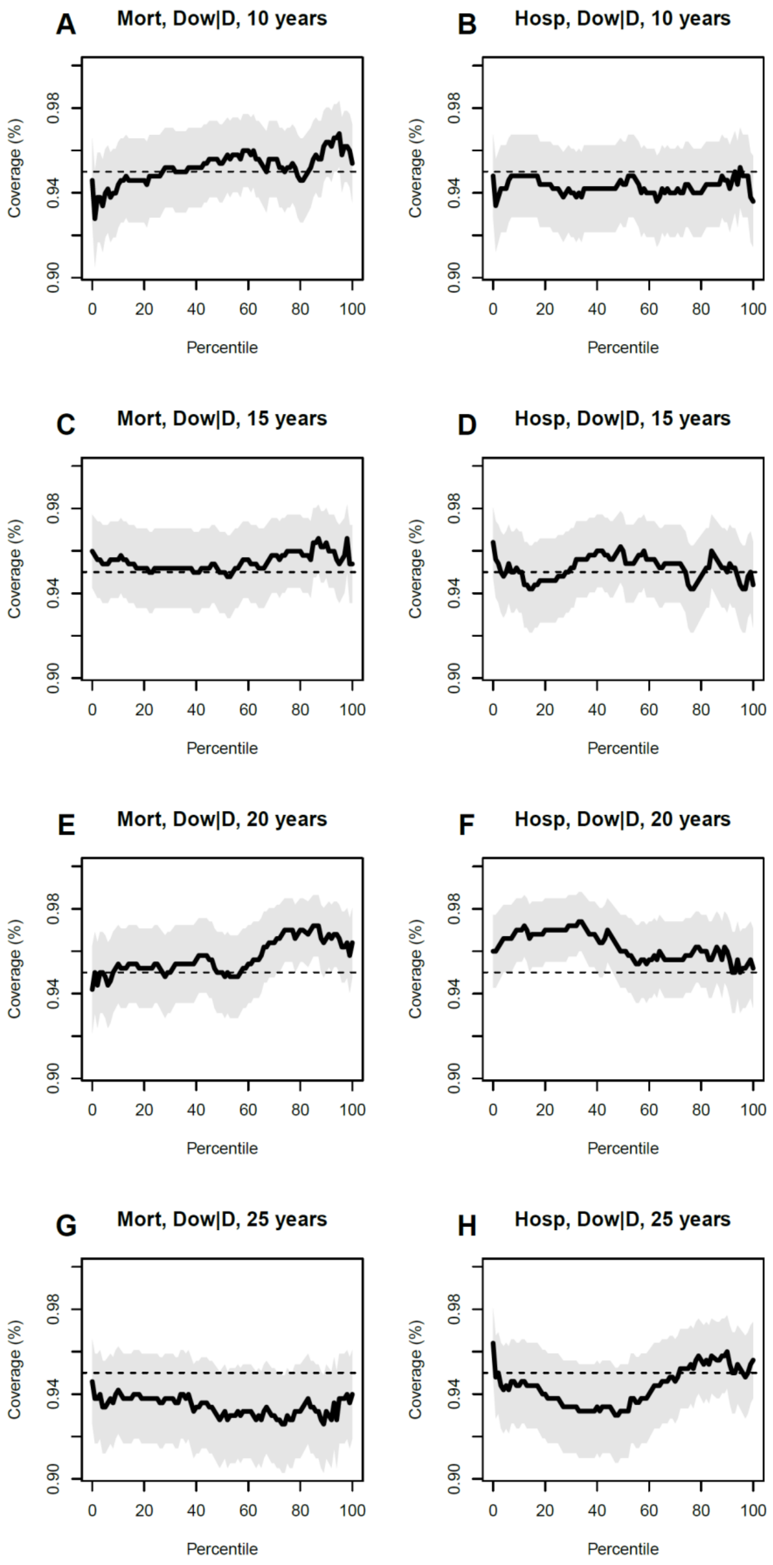

eFigure 8. Coverage of 95% confidence intervals at each temperature percentile (Dow|D model). Panels in the left column (A, C, E, G) show results obtained for mortality models, while panels in the right column (B, D, F, H) show results obtained for respiratory hospitalization models. Results for 10 years (A, B), 15 years (C, D), 20 years (E, F) and 25 years (G, H) are presented. Dashed lines correspond to the nominal 95% level. Shaded areas correspond to the 95% confidence intervals.

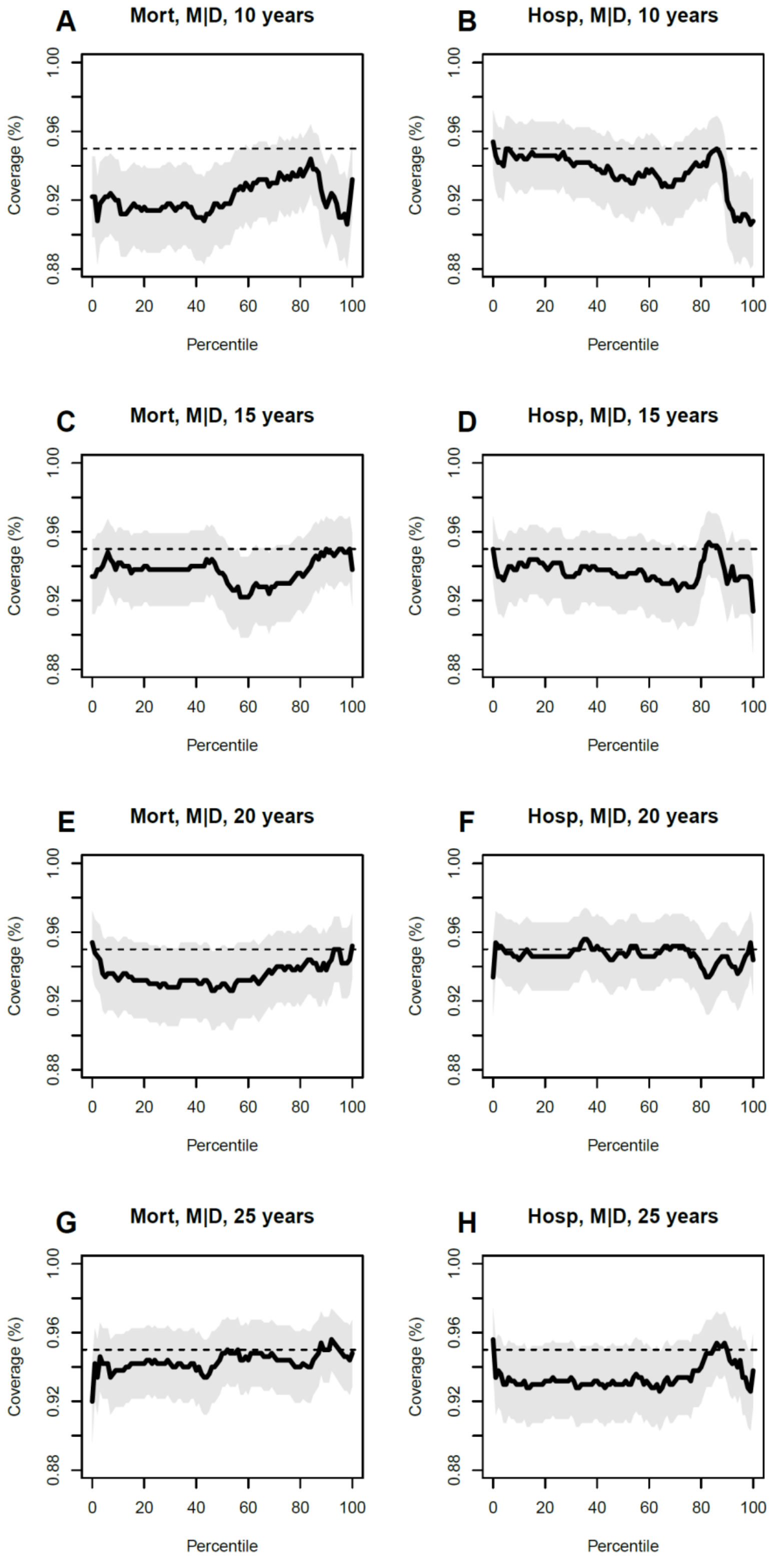

eFigure 9. Coverage of 95% confidence intervals at each temperature percentile (M|D model). Panels in the left column (A, C, E, G) show results obtained for mortality models, while panels in the right column (B, D, F, H) show results obtained for respiratory hospitalization models. Results for 10 years (A, B), 15 years (C, D), 20 years (E, F) and 25 years (G, H) are presented. Dashed lines correspond to the nominal 95% level. Shaded areas correspond to the 95% confidence intervals.

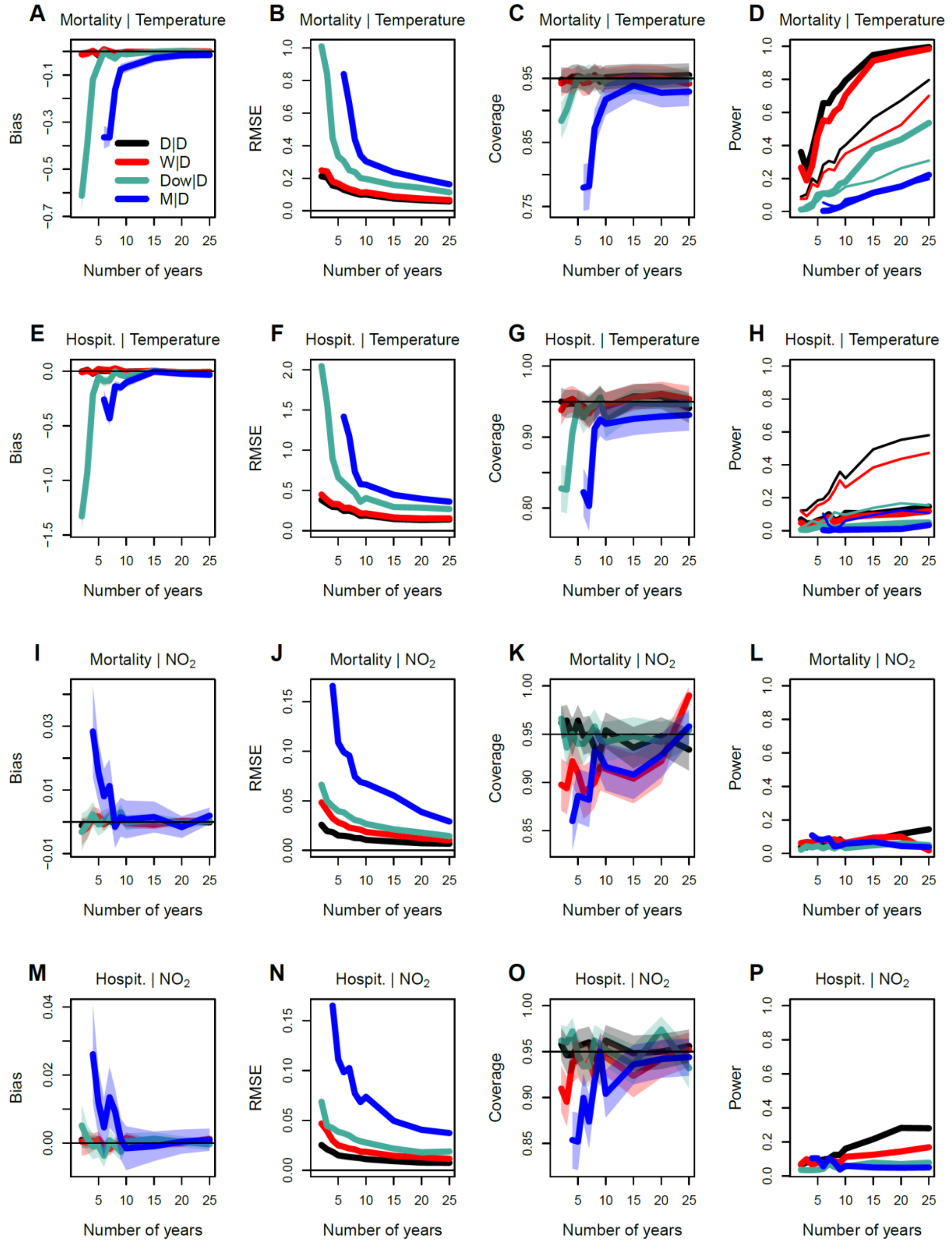

eFigure 10. Bias, root mean squared error, coverage and statistical power of the cumulative exposure-response association, for the case where the number of cases is divided by 10.
Average bias (A, E, I, M), root mean squared error (RMSE; B, F, J, N), coverage of 95% confidence intervals (C, G, K, O), and statistical power at 5% significance level (D, H, L, P) of the exposure-response association as a function of the length of the dataset. For temperature (A-H), we calculated these metrics from the cumulative exposure-response function, computed from the logarithm of the relative risks and centered at the minimum mortality temperature of the true association. We calculated the statistical power for detecting the association at the 1st (thin lines) and 99th (thick lines) percentiles of the daily temperature distribution (D,H), or for the single regression coefficient of NO_2 (L,P). Lines represent performance values in the Daily|Daily (D|D, black), Weekly|Daily (W|D, red) and Monthly|Daily (M|D, blue) models, as well as for groups of days corresponding to the same day of the week, month and year (e.g. all Mondays of January 2025; Dow|D, green). Shaded areas in the bias and coverage correspond to 95% confidence intervals.
